# Case report: Vaccine-induced immune thrombotic thrombocytopenia complicated by acute cerebral venous thrombosis and hemorrhage after AstraZeneca vaccines followed by Moderna COVID-19 vaccine booster and surgery

**DOI:** 10.3389/fneur.2022.989730

**Published:** 2022-10-04

**Authors:** Quan-Ting Chen, Yi Liu, Yeu-Chin Chen, Chung-Hsing Chou, Yu-Pang Lin, Yun-Qian Lin, Ming-Chen Tsai, Bo-Kang Chang, Tsung-Han Ho, Chun-Chi Lu, Yueh-Feng Sung

**Affiliations:** ^1^Department of Neurology, Tri-Service General Hospital, National Defense Medical Center, Taipei, Taiwan; ^2^Department of Internal Medicine, Taoyuan Armed Forces General Hospital, Taoyuan, Taiwan; ^3^Division of Hematology/Oncology, Department of Internal Medicine, Tri-Service General Hospital, National Defense Medical Center, Taipei, Taiwan; ^4^Hemophilia Care and Research Center, Tri-Service General Hospital, Taipei, Taiwan; ^5^Department of Radiology, Tri-Service General Hospital, National Defense Medical Center, Taipei, Taiwan; ^6^Division of Rheumatology/Immunology/Allergy, Department of Internal Medicine, Tri-Service General Hospital, National Defense Medical Center, Taipei, Taiwan

**Keywords:** vaccine-induced thrombotic thrombocytopenia, cerebral hemorrhage, Moderna booster, autoimmune heparin-induced thrombocytopenia, cerebral venous thrombosis

## Abstract

Vaccine-induced thrombotic thrombocytopenia (VITT) is a well-known complication of adenoviral vector COVID-19 vaccines including ChAdOx1 nCoV-19 (AstraZeneca) and Ad26. COV2.S (Janssen, Johnson & Johnson). To date, only a few cases of mRNA COVID-19 vaccine such as mRNA-1273 (Moderna) or BNT162b2 (Pfizer-BioNTech)-induced VITT have been reported. We report a case of VITT with acute cerebral venous thrombosis and hemorrhage after a booster of mRNA-1273 (Moderna) vaccine in a patient previously vaccinated with two doses of the AstraZeneca vaccine. A 42-year-old woman presented with sudden onset of weakness of the right upper limb with focal seizure. She had received two doses of AstraZeneca vaccines and a booster with Moderna vaccine 32 days before presentation. She had also undergone a laparoscopic myomectomy 12 days previously. Laboratory examinations revealed anemia (9.5 g/dl), thrombocytopenia (31 × 10^3^/μl), and markedly elevated d-dimer (>20.0 mg/L; reference value < 0.5 mg/L). The initial brain computed tomography (CT) was normal, but a repeated scan 10 h later revealed hemorrhage at the left cerebrum. Before the results of the blood smear were received, on suspicion of thrombotic microangiopathy with thrombocytopenia and thrombosis, plasmapheresis and pulse steroid therapy were initiated, followed by intravenous immunoglobulin (1 g/kg/day for two consecutive days) due to refractory thrombocytopenia. VITT was confirmed by positive anti-PF4 antibody and both heparin-induced and PF4-induced platelet activation testing. Clinicians should be aware that mRNA-1273 Moderna, an mRNA-based vaccine, may be associated with VITT with catastrophic complications. Additionally, prior exposure to the AstraZeneca vaccine and surgical procedure could also have precipitated or aggravated autoimmune heparin-induced thrombocytopenia/VITT-like presentation.

## Introduction

Coronavirus disease 2019 (COVID-19) has caused six million deaths globally since 2019, the majority with severe respiratory complications. Several vaccines against SARS-CoV-2 were developed and administered worldwide, including ChAdOx1 nCoV-19 (AstraZeneca), BNT162b2 (BioNTech/Pfizer), mRNA-1273 (Moderna), and Ad26.COV2.S (Janssen; Johnson & Johnson). Despite the high efficacy of these vaccines, the virus developed different variants, such as the omicron variant, making the vaccines less effective. To increase protection, a booster shot after two injections and combined multiple source-based vaccines were suggested. While these strategies were intended to improve immunity, they could also increase the risk of adverse effects.

Vaccine-induced immune thrombotic thrombocytopenia (VITT) was first reported by Greinacher et al. and proposed a pathophysiology resembling that of heparin-induced thrombocytopenia/thrombosis (1). The pathogenesis of these thrombotic events involves the generation of antibodies that bind to platelet factor 4 (PF4), resulting in platelet activation, aggregation, and thrombosis formation. Treatment strategies include anticoagulation, preferably with a non-heparin agent, correction of low fibrinogen with cryoprecipitate, consideration of intravenous immunoglobulin (IVIG), steroids, and plasmapheresis (2). In a prospective cohort study, patients with VITT who received AstraZeneca vaccines have a mortality rate of approximately 22%, which increased to 2.7 times among patients with cerebral venous thrombosis. The mortality associated with VITT was the highest among patients with a low platelet count and intracranial hemorrhage (3).

An association between adenoviral vector-based vaccines and VITT is well recognized. However, there is little to no information in the literature about VITT after receiving an mRNA vaccine such as the Moderna vaccine, or more precisely, VITT after a booster of the Moderna vaccine following previous exposure to the adenoviral AstraZeneca vaccine, which raises the possibility of a cumulative effect of induction of thrombogenic autoimmunity. Herein, we present such a case of VITT complicated by cerebral venous thrombosis and hemorrhage.

## Case

A 42-year-old woman presented to the emergency room with sudden onset of weakness and numbness of the right upper limb. Her past medical history was unremarkable except for occasional headaches, which were related to her menstrual cycle and stress. She had been suffering from menorrhagia and dysmenorrhagia for a year, and a huge uterine myoma (9.3 × 10.2 × 7.7 cm over right posterior wall) was found. She only took iron supplements and had not used oral contraceptives before. She had received the first and second doses of the ChAdOx1 nCoV-19 (AstraZeneca) vaccine seven and 4 months before hospitalization, and she received a booster dose of the Moderna vaccine 32 days before the onset of symptoms. Of note, 12 days before the presentation, she had a laparoscopic myomectomy under general anesthesia. The medications used in anesthesia included fentanyl, lidocaine, propofol, rocuronium bromide, dexamethasone, glycopyrrolate, and desflurane inhalation. Her vital signs before, during, and after surgery were normal. She reported mild dizziness after surgery. Two days after surgery, she began to experience a mild headache, nausea, and lethargy. Frequent vaginal bleeding was also noted, but the amount of bleeding was small. The hemoglobin (15.3 g/dl; reference value 12–16 g/dl), platelet count (121 × 10^3^/μl; reference value, 150–400 × 10^3^/μl), coagulation parameters, and blood biochemistry tests were normal before the surgery. However, on the first postoperative day, she was found to be anemic (Hgb 8.2 g/dl) and thrombocytopenic (platelets 121 × 10^3^/μl). The rapid decline of hemoglobin was thought to be due to blood loss (about 400 ml during surgery) and hemodilution (fluid replacement with colloid 500 ml and crystalloid 1,100 ml during surgery, and dextrose 5% in water 1,000 ml and 0.9% normal saline 1,000 ml after surgery). The platelet count further decreased to 31 × 10^3^/μl 8 days later in the gynecology outpatient department visit. There was no heparin exposure, infection, or blood transfusion during that hospitalization.

On her current admission to the hospital, her body temperature was 36.8°C; blood pressure, 125/94 mmHg; heart rate, 96 beats per minute; respiratory rate, 22 times per minute. Physical and neurological examinations revealed mild pale conjunctiva, weakness of the right hand (Medical Research Council scale grade 3), and paresthesia of the right upper limb. The National Institutes of Health Stroke Scale score was 2. Laboratory studies showed anemia (9.5 g/dl), thrombocytopenia (31 × 10^3^/μl), and elevated D-dimer (>20.0 mg/L; reference value <0.5 mg/L). Brain computed tomography (CT) without contrast enhancement revealed no remarkable findings. Antiplatelet treatment with aspirin 300 mg was orally administered on suspicion of acute ischemic stroke. Focal-onset aware seizure of the right upper and lower limbs was observed. The repeated brain CT still showed no abnormalities. Levetiracetam 1,500 mg was administered intravenously on suspicion of early poststroke seizure.

**Figure 1:**
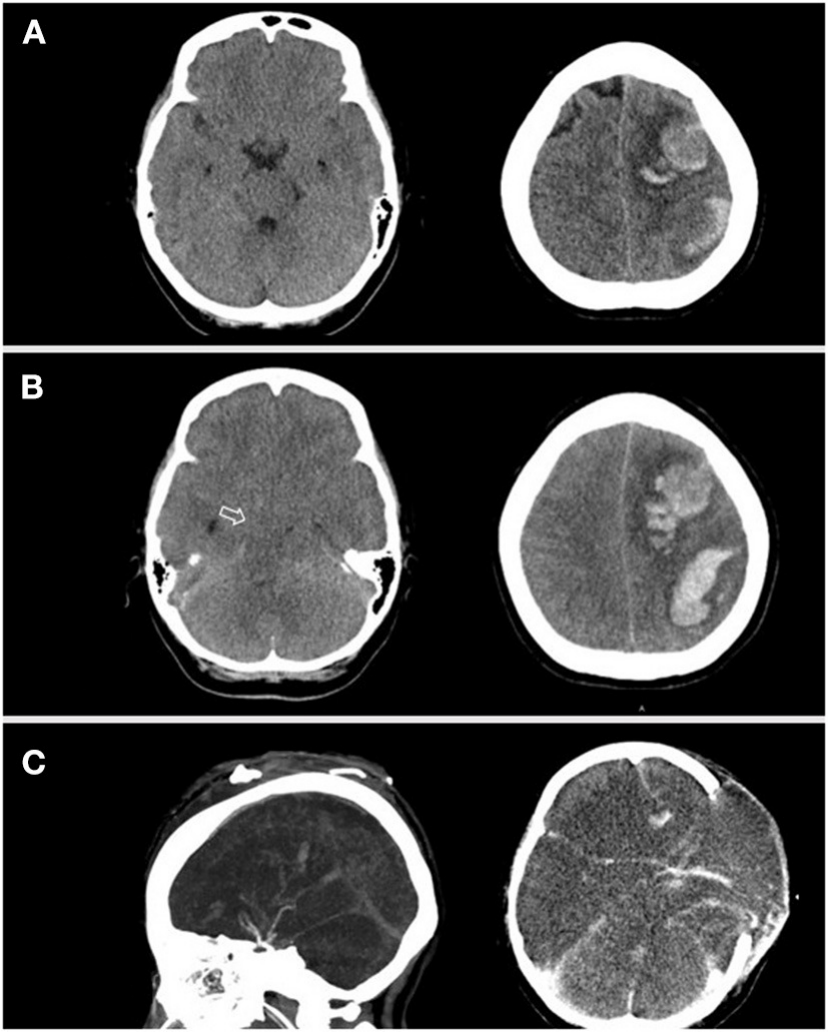
A series of brain CT over the disease course. **(A)** Brain CT revealed two lobar hemorrhages located in the left frontal and parietal lobes with perifocal edema. **(B)** Five hours later, the patient's brainstem reflex was lost, and brain CT showed progressive hemorrhage with diffuse cerebral edema and bilateral uncal herniation (arrow). **(C)** Contrast-enhanced brain CT obtained 5 days after craniectomy showed suboptimal vascular image quality due to prominent intracranial hypertension.

**Figure 2:**
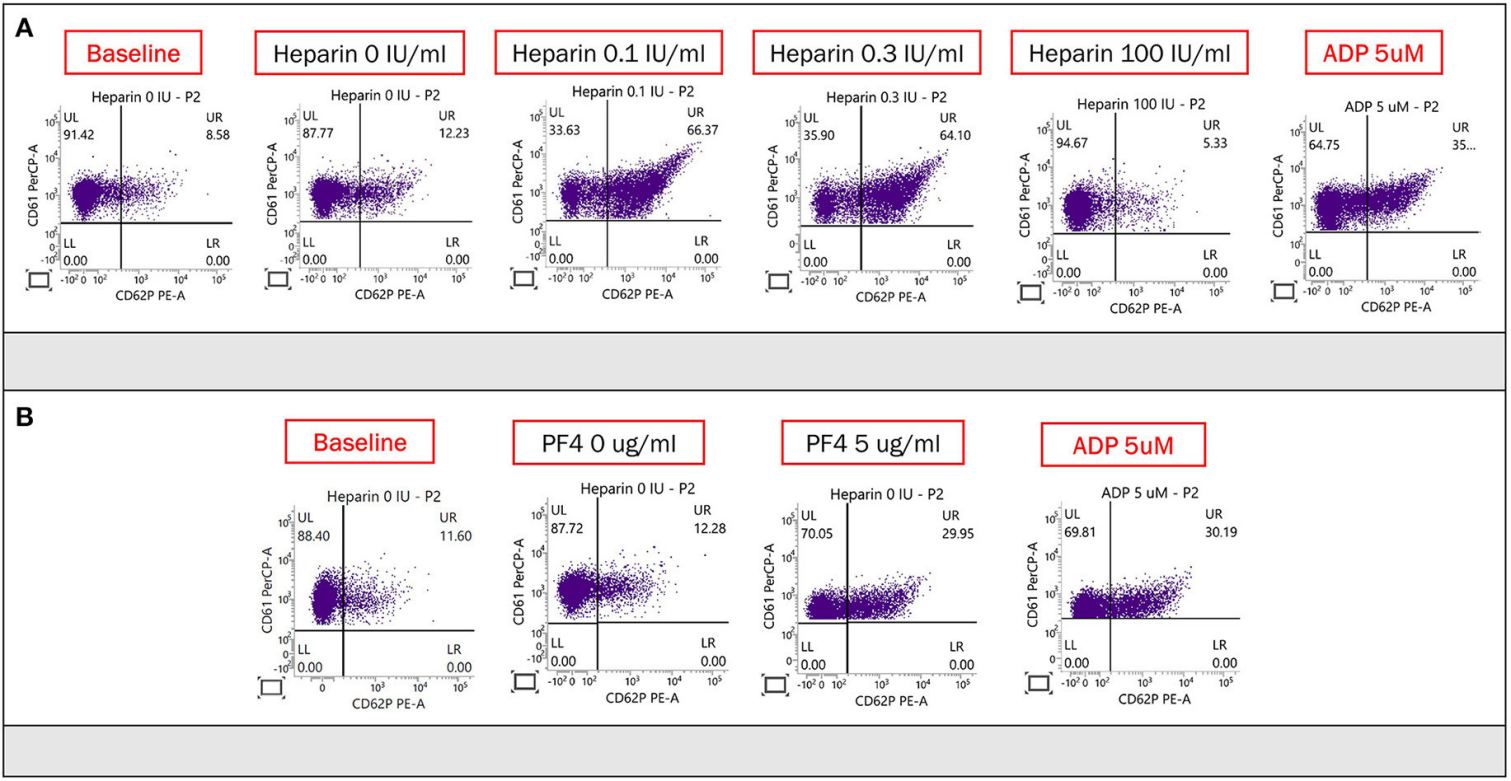
**(A)** Heparin-induced platelet activation assay was used to detect of HIT antibodies. CD61 (glycoprotein IIIa) and CD62p (p-selectin) served as markers of platelet identification and activation, respectively. Adenosine diphosphate was used to confirm normal platelet activation. The proportion of activated platelets was at least >11% in the presence of heparin (0.1 or 0.3 IU/ml) compared with baseline (no heparin), and the activation could be suppressed by a high dose of heparin (100 IU/ml). There was obvious platelet activation in the presence of the patient's plasma and low concentration (0.1 and 0.3 U/ml) of heparin, which was suppressed by the high concentration of heparin (100 U/ml). **(B)** PF4-induced flow cytometry-based platelet activation (PIFPA) revealed that the percentage of activated platelets increased from 12.28% baseline, no PF4 addition) to 29.95% with addition of 5 μg/ml PF4.

Ten hours after admission, she had progression of right-sided weakness and consciousness change. Brain CT revealed two lobar hemorrhages over the left frontal and parietal lobes with perifocal edema and mild midline shift (Figure 1A). She was intubated immediately. On suspicion of VITT or other autoimmune disorder-related thrombocytopenia such as catastrophic antiphospholipid syndrome, systemic lupus erythematosus (SLE) with central nervous system involvement, and thrombotic thrombocytopenic purpura, plasmapheresis was arranged, and methylprednisolone 1 g/day was administered intravenously. Unfortunately, her consciousness deteriorated rapidly, declined from E2M4VT to E1M1VT, and bilateral pupils were dilated to 8 mm without light reflexes. The follow-up brain CT revealed rapid expansion of hematoma with marked brain edema, midline shift, and uncal and tonsillar herniation. The lobar hemorrhage extended into the subdural and subarachnoid spaces (Figure 1B). Emergent decompressive craniectomy was performed after transfusion of two units of single-donor platelets. Plasmapheresis was undertaken immediately after the surgery. The intracranial cerebral pressure (ICP) increased to 120 mmHg on day 3 of admission, despite the use of mannitol, glycerol, 3% normal saline, and mechanical hyperventilation. The enzyme-linked immunosorbent assay for anti-PF4 polyanion antibody was positive (test value, 2.91 optical density; reference value, < 0.4 optical density). ADAMTS-13 activity was normal, and markers of SLE or anti-phospholipid syndromes were all negative (Supplementary material). The blood smear was negative for schistocytes. The result of the platelet activation assay was consistent with VITT (Figure 2) thereby confirming the diagnosis of VITT.

**Figure 3:**
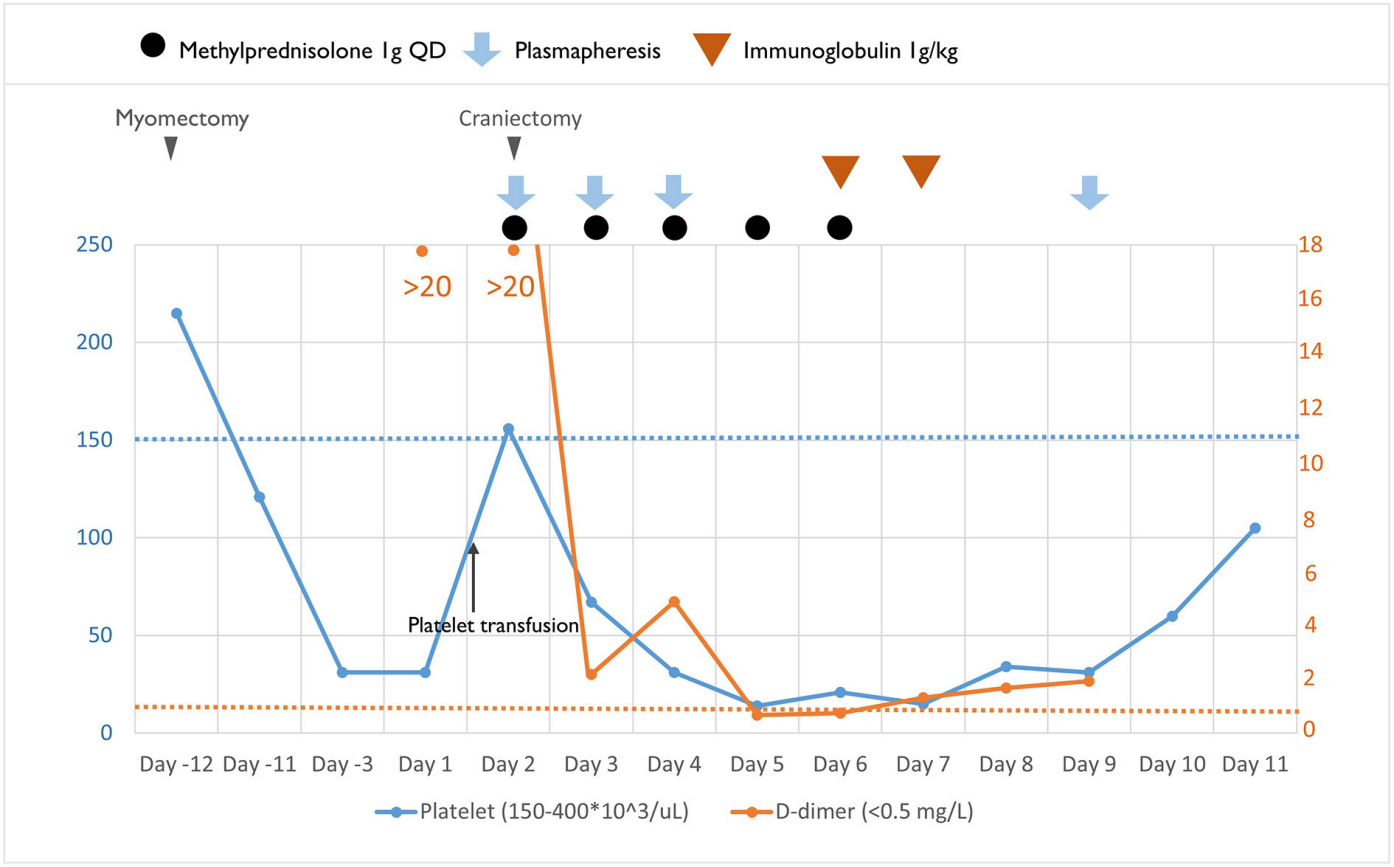
Clinical course, the laboratory studies and therapeutic agents used.

No improvement in thrombocytopenia was observed after 3 days of plasmapheresis (days 2, 4, and 5 of admission, Figure 3). We therefore switched to immunoglobulin (1 g/kg/day) administered intravenously for two consecutive days on days 5 and 6 of admission. However, platelet count remained low (16–34 × 10^3^/μl), and plasmapheresis was restarted on day 9 of admission. The platelet count improved to 60 × 10^3^/μl and 105 × 10^3^/μl on days 10 and 11 of admission, respectively (Figure 3). Although the patient's peripheral arterial oxygen saturation was maintained at 99%−100% before, during, and after surgery, head CT on day 6 showed diffuse brain edema and loss of gray-white matter junction, involving the bilateral cerebrum, basal ganglia, brain stem, and cerebellum with obliteration of all ventricles (Figure 1C). The findings were indicative of diffuse hypoxic ischemic brain injury, which could be a result of decreased brain perfusion secondary to increased ICP. Autonomic dysfunction, arrhythmia, refractory shock, and central diabetes insidious with hypernatremia developed subsequently. Because of irreversible severe brain injury, her family decided on hospice care with withdrawal of ventilatory support on day 11 of admission, and the patient expired.

## Discussion

We present a 42-year-old woman with VITT about a month following a Moderna COVID-19 vaccine booster, complicated by catastrophic cerebral venous thrombosis, intracranial hemorrhage, and uncal herniation, eventually leading to the patient's demise. To the best of our knowledge, there are no previous reports of VITT complicated with cerebral venous thrombosis and hemorrhage associated with the Moderna vaccine. Elevated anti-PF4 antibodies are not specific for VITT diagnosis, so we performed the heparin-induced platelet activation assay. This assay can be positive in both HIT and VITT (4). We therefore performed PF4-induced platelet activation by flow cytometry-based assay for confirmatory diagnosis of VITT. This can distinguish VITT from HIT because unlike HIT which requires the presence of heparin, platelet activation in VITT occurs in the presence of PF4 alone (5).

VITT is a rare, but severe complication of COVID-19 vaccines. Most cases were related to the AstraZeneca vaccine, and only few cases were reported to be related to the Moderna vaccine (6–8). According to the American Society of Hematology (2), the diagnosis of VITT must meet all five criteria including (1) COVID vaccination 4–42 days before symptom onset, (2) any venous or arterial thrombosis (often cerebral or abdominal location), (3) thrombocytopenia (platelet count < 150 × 10^9^/L), (4) positive anti-PF4 antibody, and (5) markedly elevated D-dimer (more than four times the upper limit of normal). Our patient met all these diagnostic criteria of VITT. A CT venography (CTV) was not performed initially, and the subsequent severe cerebral swelling precluded the assessment of venous filling defect by CTV (9). Because of the use of postoperative staples on the scalp and infusion pump from the second day after admission, the patient had no chance to receive brain magnetic resonance imaging. However, the preceding nausea, headache, followed by focal neurologic deficit with seizure imply the high probability of cerebral venous thrombosis (CVT) rather than arterial thrombosis, and the location of cerebral hemorrhage at juxtacortical white matter is suggestive of CVT with hemorrhagic transformation (10).

In this patient, the onset time of neurological symptoms was 32 days after the Moderna vaccination. Moderna vaccine-induced thrombocytopenic petechiae/purpura has been reported previously (8); however, our patient developed more life-threatening complications, including CVT and intracranial hemorrhage with rapid progression of brain edema and uncal herniation. The patient had undergone laparoscopic myomectomy 12 days before presentation and experienced mild headache, nausea, and lethargy after the surgery. In addition, mild thrombocytopenia was observed 1 day after the surgery. Postoperative headaches are not uncommon in clinical practice, and postoperative thrombocytopenia can be caused by hemodilution and consumption. In our patient, spontaneous HIT, which is a subtype of HIT without preceding heparin exposure, could also explain a clinical and serologic picture similar to VITT. Spontaneous HIT has been largely reported after orthopedic surgery and some other exposures such as polyanionic medications or virus/bacterial infection (11). Occasionally, no preceding trigger is identified (12). From the available data in our patient, it was not possible to definitively determine if spontaneous HIT induced by laparoscopic myomectomy was the only cause of her clinical presentation with acute thrombosis or a precipitating factor for VITT development from prior exposure to the Moderna vaccine booster. It is also uncertain whether there may have been persistent low-level/subclinical HIT-like antibodies from more remote AstraZeneca vaccine exposure that may have added to the overall cumulative risk of thrombosis.

Once a diagnosis of VITT is established, treatment involves (1) IVIG 1 g/kg daily for 2 days, (2) non-heparin anticoagulant agents, (3) avoiding platelet transfusions, (4) corticosteroids that do not have sufficient data to prove their role, (5) avoiding aspirin, since it does not help with treatment or prophylaxis and may increase bleeding risk, and (6) plasmapheresis, which is an additional option when thrombosis progresses despite IVIG and non-heparin anticoagulants (13). Because VITT was not suspected initially in our patient, one dose of 300 mg aspirin was administered orally at the emergency room under the impression of acute ischemic stroke. For CVT, heparin is the standard treatment, but is best avoided in VITT cases. The safer anticoagulant for VITT is direct oral anticoagulant (DOAC). In our patient, the cerebral hemorrhage could have been a complication of aspirin or CVT. Unfortunately, the rapid expansion of the hematoma precluded the use of DOAC.

The initial uncertain diagnosis and a rapid and catastrophic course in our case led us to choose plasmapheresis and steroid pulse therapy first as broad coverage of VITT and other potential autoimmune diseases that were in the differential, such as catastrophic antiphospholipid syndrome, SLE with central nervous system involvement, and thrombotic thrombocytopenic purpura (14). Previous studies have suggested good efficacy of plasmapheresis for patients with VITT who have thrombocytopenia refractory to IVIG therapy (14, 15). In our patient, thrombocytopenia was not responsive to the initial three treatments of plasmapheresis. Partial improvement in thrombocytopenia was observed after 2 days of IVIG and another treatment of plasmapheresis. It appears that plasmapheresis overall was not effective in our patient, or possibly, the effect of plasmapheresis for thrombocytopenia was delayed.

## Conclusion

While VITT is typically caused by adenovirus-based vaccines, our case highlights the possibility of the Moderna vaccine, a messenger RNA-based vaccine, as a potential precipitant of VITT in a patient with remote exposure to an adenoviral vaccine. The prior AstraZeneca vaccine exposure and the gynecologic surgical procedure before and after the Moderna vaccination respectively could have cumulatively precipitated or aggravated autoimmune HIT/VITT-like presentation. During the COVID-19 pandemic, awareness of this possibility could allow clinicians to consider VITT as a potential diagnosis in patients who present with thrombosis and low platelets and have received a COVID-19 vaccination, even if the time frame and vaccine type are not typical for VITT. Post-surgery headaches are common; however, since they are an early sign of increased intracranial pressure caused by CVT it is challenging for clinicians to identify the latter early based on this symptom alone. Generally, antiplatelet therapy should be started in patients with acute stroke as soon as possible after brain imaging has excluded hemorrhage. However, clinicians should look at laboratory data, evaluate the patient's past medical history, and consider other stroke mimics thoroughly before antiplatelets are given. In cases of VITT, aspirin is not recommended because of the increased bleeding risk. A high index of suspicion in such cases could facilitate early diagnosis, which could lead to timely and aggressive intervention, and in turn may help to prevent severe morbidity or mortality.

## Data availability statement

The raw data supporting the conclusions of this article will be made available by the authors, without undue reservation.

## Ethics statement

Written informed consent was obtained from the patients for the publication of any potentially identifiable images or data included in this article.

## Author contributions

Y-CC, C-CL, and Y-FS contributed to the conception and design of the study. C-HC, Y-QL, M-CT, B-KC, Y-PL, and T-HH contributed to the acquisition and analysis of data. Q-TC, YL, Y-CC, and Y-FS contributed to drafting the text or preparing the figures. All authors contributed to the article and approved the submitted version.

## Conflict of interest

The authors declare that the research was conducted in the absence of any commercial or financial relationships that could be construed as a potential conflict of interest.

## Publisher's note

All claims expressed in this article are solely those of the authors and do not necessarily represent those of their affiliated organizations, or those of the publisher, the editors and the reviewers. Any product that may be evaluated in this article, or claim that may be made by its manufacturer, is not guaranteed or endorsed by the publisher.
