# Supplementary material for: Case report: Vaccine-induced immune thrombotic thrombocytopenia complicated by acute cerebral venous thrombosis and hemorrhage after AstraZeneca vaccines followed by Moderna COVID-19 vaccine booster and surgery
Source: Front Neurol. 2022 Oct 4;13:989730. doi: 10.3389/fneur.2022.989730 (PMC9577219; doi:10.3389/fneur.2022.989730)
Supplement: Supplementary file 1 [file Table_1.docx]

Supplementary Material

Table 1

| **Items** | **Results** | **Normal range** |
| --- | --- | --- |
| **WBC** | 4.56 | 4.5-11* 10^3/uL |
| **Hb** | **9.5** | 12-16 g/dL |
| **MCV** | 96.2 | 79-100 fL |
| **PLT** | **31** | 150-400*10^3/uL |
| **Na** | 138 | 136-145mmol/L |
| **K** | 3.9 | 3.5-5.1 mmol/L |
| **BUN** | 8 | 7─25 mg/dL |
| **creatinine** | 0.6 | 0.5-0.9 mg/dL |
| **AST** | 13 | <40 U/L |
| **ALT** | 14 | <41 U/L |
| **Free Calcium** | 4.55 | 4.5-5.3 mg/dL |
| **Magnesium** | 2.4 | 1.7─2.55mg/dL |
| **SICV2(RT-PCR)** | Negative |  |
| **PT (INR)** | 13.3 (1.0) | 11-15 sec |
| **APTT** | 39 | 29.6─46.2 |
| **D-dimer** | **>20** | <0.5 mg/L(FEU) |
| **CK** | 41 | 26-192 U/L |
| **Troponin I** | <2 | <40 pg/mL |
| **Total Bilirubin** | 0.6 | 0.3-1 mg/dL |
| **Direct Bilirubin** | 0.1 | < 0.2 mg/dL |
| **Lactate** | 1.8 | 0.5-2.2 mmol/L |
| **Homocysteine** | **3.50** | 5─15 umol/L |
| **RPR** | Non-reactive |  |
| **HDL** | 41 | >55 mg/dl |
| **LDL** | 110 | <100 mg/dL |
| **Albumin** | 3.4 | 3.5─5.7 g/dL |
| **Total Cholesterol** | 168 | 200 mg/dL |
| **Vitamin B12** | 518 | 197-771 pg/mL |
| **Folate** | 26.8 | 4.8-37.3 ng/mL |
| **HIV Ag/Ab** | Negative |  |
| **Hematologic profiles** | | |
| **Reticulocyte Count** | **4.32** | 0.50─1.50 % |
| **Immunoglobulin G** | 880 | 635─1741 mg/dL |
| **Immunoglobulin M** | 128 | 45─281 mg/dL |
| **Immunoglobulin A** | 320 | 66─433 mg/dL |
| **Cryoglobulin** | Negative |  |
| **ASOT** | <100 | < 250 IU/mL |
| **LDH** | 217 | 140-271 U/L |
| **Haptoglobin** | **<30.0** | 44-215 mg/dL |
| **H. pylori Ag** | Negative |  |
| **CMV PCR** | Negative |  |
| **EBV viral load** | Not detected |  |
| **Rheumatologic profiles** | | |
| **Anti-beta 2-Glycoprotein I Ig** | <0.6 | <10.0 U/ml |
| **Anti-Cardiolipin IgG** | 16.00 | < 40 GPL-U/ml |
| **Anti-Cardiolipin IgM** | 0.9 | < 40 MPL-U/ml |
| **Lupus anticoagulant (DRVVT)** | 1.01 | <1.2 |
| **Anti-ds DNA** | 0.6 | 0.0-15.0 IU/ML |
| **ANA** | Negative |  |
| **ANCA** | Negative |  |
| **Rheumatoid Factor** | <10.0 | < 14 IU/mL |
| **C3** | 135.6 | 87-200 mg/dL |
| **C4** | 34.7 | 19-52 mg/dL |
| **Anti-PF4 (day 1)** | **2.91** | < 0.39 OD |
| **Anti-PF4 (day 6)** | **0.6** | <0.4 OD* |
| **Anti-PF4 (day 7)** | **1.82** | < 0.39 OD |
| *Results from Center for Tissue Engineering, Chang Gung Memorial Hospital | | |

Table 2

|  | **Day 1** | **Day 2** | **Day 3** | **Day 4** | **Day 5** | **Day 6** | **Day 7** | **Day 8** | **Day 9** | **Day 10** |
| --- | --- | --- | --- | --- | --- | --- | --- | --- | --- | --- |
| **Fibrinogen (200-400mg/dL)** | - | - | - | 292.0 | 328.9 | 262.1 | 266.7 | **173.3** | **196.6** | **167.8** |
| **Protein C**  **(70-140%)** | - | 96.4 | - | - | - | 97 | **160.6** | **176.9** | **163.8** | **175.0** |
| **Protein S**  **(52.1-126%)** | - | 68- | - | - | - | 58.2 | 100.6 | 94.7 | 98.9 | 92.3 |
| **Intracranial pressure**  **(<20 mmHg)** | - | **54** | **112** | **70** | **63** | **37** | **56** | - | - | - |
